# Supplementary material for: Atrial Fibrillation Progression Is Associated with Cell Senescence Burden as Determined by p53 and p16 Expression
Source: J Clin Med. 2019 Dec 23;9(1):36. doi: 10.3390/jcm9010036 (PMC7019631; doi:10.3390/jcm9010036)
Supplement: Supplementary file 1 [file jcm-09-00036-s001.pdf]

Supplementary Table 1. Predictors of TF elevation: univariate and multivariate analysis.

	Univariate Analysis			Multivariate Analysis		
	**HR**	**95% CI**	***p***	**HR**	**95% CI**	***p***
Age	0.986	0.935–1.040	0.603			
AF	2.641	0.760–9.176	0.127			
Permanent AF	1.700	0.402–7.198	0.471			
Paroxysmal AF	1.600	0.413–6.193	0.496			
AF at the Time of Surgery	2.167	0.631–7.442	0.129			
Hypertension	0.533	0.148–1.922	0.336			
Diabetes Mellitus	0.813	0.229–2.877	0.748			
Smoking	0.417	0.111–1.567	0.195			
Female Sexe	1.083	0.288–4.081	0.906			
LVEF < 40%	188.47	0.001–487.243	0.999			
CHA2DS2VASc	0.921	0.645–1.315	0.652			
Statins	1.038	0.226–4.768	0.961			
ACEinhibitor/AT1blocker	0.440	0.13–1.577	0.208			
Coronaropathy	0.677	0.198–2.312	0.534			
Euroscore I	1.061	0.918–1.227	0.424			
Euroscore II	1.109	0.871–1.412	0.401			
LA area	1.018	0.968–1.071	0.496			
p16	1.376	0.211–8.971	0.738			
p 53	18.091	2.214–147.820	0.007			

AF atrial fibrillation; LVEF left ventricle ejection fraction; LA left atrium.

Supplementary Table 2. Predictors of eNOS elevation: univariate and multivariate analysis.

	Univariate Analysis			Multivariate Analysis		
	**HR**	**95% CI**	***p***	**HR**	**95% CI**	***p***
Age	1.038	10.981–1.098	0.194			
AF	0.231	0.061–0.869	0.030			
Permanent AF	0.328	0.071–1.518	0.154			
Paroxysmal AF	0.583	0.136–2.498	0.468			
AF at the Time of Surgery	0.290	0.079–1.063	0.062			
Hypertension	1.556	0.420–5.763	0.508			
Diabetes Mellitus	1.909	0.520–7.007	0.330			
Smoking	0.407	0.106–1.559	0.190			
Female Sex	0.714	0.179–2.843	0.633			
LVEF < 40%	0.474	0.039–5.688	0.556			
CHA2DS2VASc	1.297	0.890–1.890	0.175			
Statins	2.800	0.570–13.754	0.205			
ACEinhibitor/AT1blocker	1.000	0.273–3.667	1.000			
Coronaropathy	1.560	0.429–5.248	0.526			
Euroscore I	0.915	0.785–1.065	0.251			
Euroscore II	0.875	0.679–1.128	0.302			
LA Area	0.984	0.939–1.032	0.515			
p16	0.128	0.012–1.372	0.089			
p 53	0.186	0.028–1.247	0.083			

AF atrial fibrillation; LVEF left ventricle ejection fraction; LA left atrium.

Supplementary Table 3. Predictors of MMP-9 elevation: univariate and multivariate analysis.

	Univariate Analysis			Multivariate Analysis		
	HR	95% CI	*p*	HR	95% CI	*p*
Age	1.092	1.017–1.173	0.015	1.071	0.995–1.154	0.069
AF	1.210	0.360–4.062	0.758			
Permanent AF	3.000	0.655–13.747	0.157			
Paroxysmal AF	0.625	0.161–2.419	0.496			
AF at the Time of Surgery	1.467	0.434–4.951	0.537			
Hypertension	8.000	1.790–35.744	0.006	5.359	1.106–25.953	0.037
Diabetes Mellitus	2.909	0.777–10.887	0.113			
Smoking	0.417	0.111–1.567	0.195			
Female Sex	2.327	0.600–9.028	0.222			
LVEF < 40%	2.105	0.176–25.170	0.556			
CHA2DS2VASc	1.980	1.246–3.146	0.004			
Statins	4.083	0.818–20.376	0.086			
ACEinhibitor/AT1blocker	0.667	0.190–2.334	0.526			
Coronary Artery Disease	1.477	0.432–5.046	0.534			
Euroscore I	1.187	0.995–1.417	0.057			
Euroscore II	1.028	0.821–1.288	0.810			
LA Area	1.049	0.976–1.126	0.192			
p16	1.258	0.194–8.171	0.810			
p 53	3.997	0.664–24.068	0.130			

AF atrial fibrillation; LVEF left ventricle ejection fraction; LA left atrium. CHA2DS2VASc score was not entered into the multivariate analysis model since this score comprises age and arterial hypertension. .
